# Fatal gastrointestinal bleeding due to IgA vasculitis complicated with tuberculous lymphadenitis: A case report and literature review

**DOI:** 10.1002/ccr3.2938

**Published:** 2020-07-06

**Authors:** Norihito Yamauchi, Shuji Tanda, Saori Kashiwagi, Atsunori Ohnishi, Munehiro Kugai, Takako Akazawa, Tsuguhiro Matsumoto, Junko Yamauchi, Akira Muramatsu, Soutaro Fujimoto

**Affiliations:** ^1^ Department of Gastroenterology Akashi City Hospital Akashi Japan; ^2^ Department of Nephrology Akashi City Hospital Akashi Japan

**Keywords:** gastrointestinal bleeding, Henoch‐Schönlein purpura, IgA vasculitis, leukocytoclastic vasculitis, tuberculosis

## Abstract

We report a case of IgA vasculitis that developed during the treatment of tuberculosis. Patients with tuberculosis who are on antituberculosis treatment can be administered steroids for severe disease or complications.

## Graphical abstract

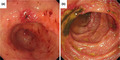

## 1 INTRODUCTION

Immunoglobulin A vasculitis (IgAV), formerly called Henoch‐Schönlein purpura, is a small vessel vasculitis that commonly occurs in children but can also affect people of all ages. Patients with this condition typically exhibit lower extremity purpura, arthritis, and hematuria. The gastrointestinal tract is affected in approximately one‐half of patients with IgAV. Gastrointestinal symptoms include abdominal pain, nausea, vomiting, and occult or overt intestinal bleeding. The condition is generally self‐limiting, and patients are usually managed with symptomatic treatment, but there are also severe cases with gastrointestinal bleeding, intussusception, infarction, and perforation.^1^, ^2^, ^3^ Although various infectious and chemical triggers are recognized, the underlying cause of IgAV remains unknown to date.^4^

We report the case of a patient with IgAV that developed during treatment of tuberculous lymphadenitis and later proved fatal because of deterioration of gastrointestinal lesions.

## 2 CASE REPORT

**Figure 1:**
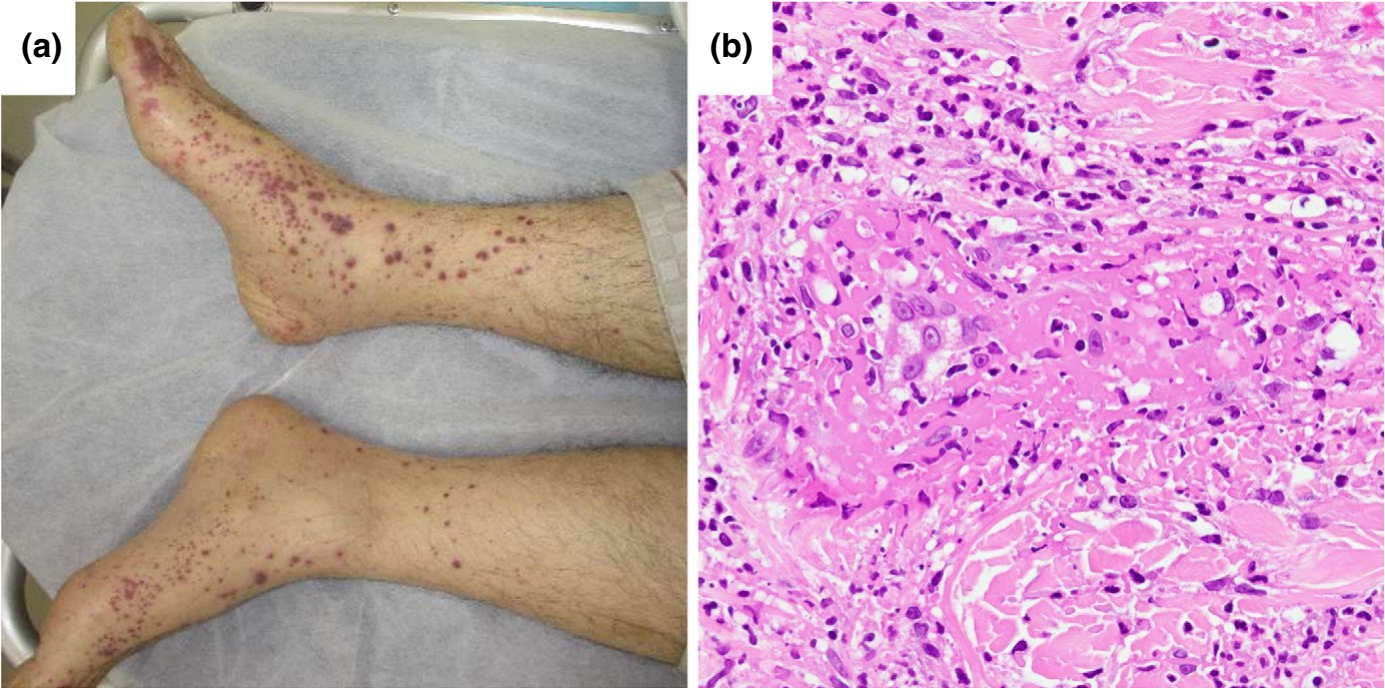
A, Palpable purpura developed on the lower extremities. B, Biopsy of skin lesions shows leukocytoclastic vasculitis with neutrophilic infiltration of small vessels in the superficial dermis. (Hematoxylin and eosin, ×400)

**Figure 2:**
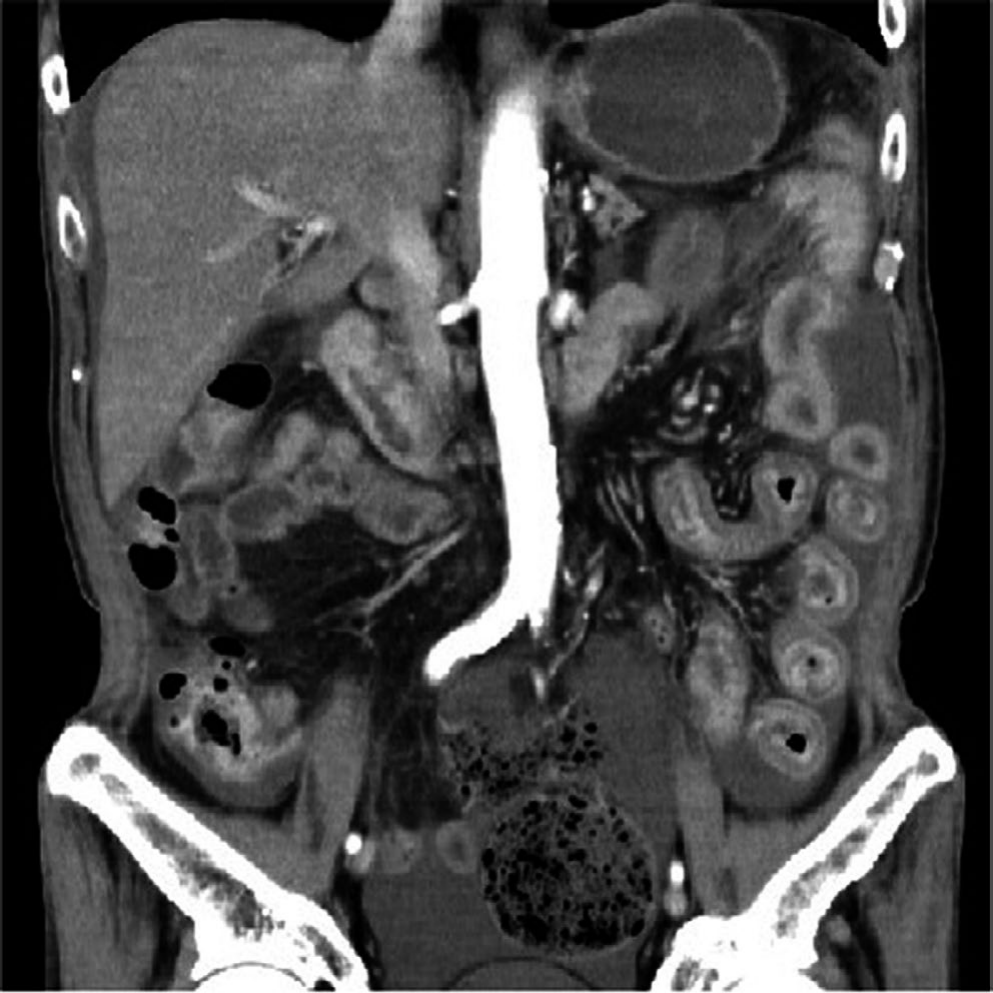
Abdominal computed tomography shows wall thickening of the duodenum and small intestine, and mild to moderate ascites

**Figure 3:**
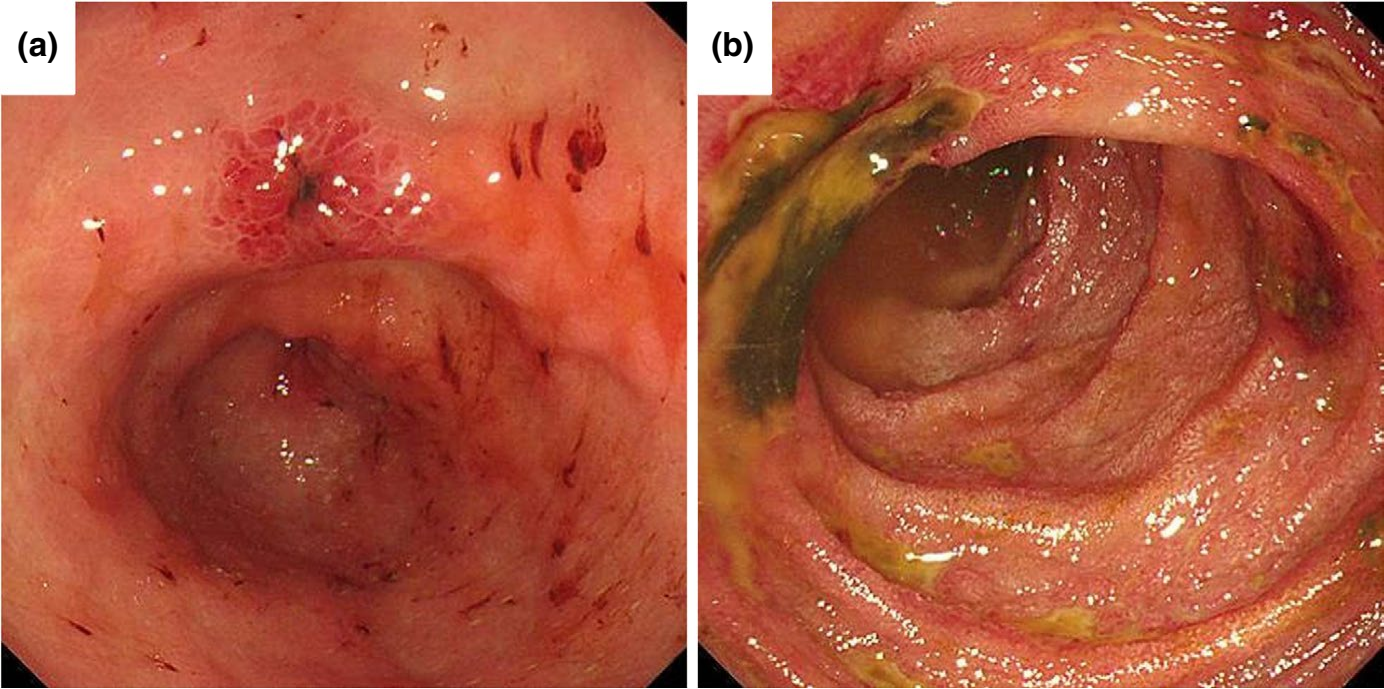
Upper gastrointestinal endoscopy shows highly reddish erosions in the antrum of the stomach (A) and circular ulcers with irregularly shaped margin in the descending duodenum (B)

A 71‐year‐old man with diabetes mellitus presented to our institution complaining of left axillary lymphadenopathy, which lasted for 1 month. Whole‐body CT revealed lymphadenopathy with central necrosis in the left clavicle, axilla, and abdomen. Scars and nonspecific fibrosis were observed in the apex of both lungs, but no active lesions or tumors were found in the lungs or other organs. *Mycobacterium tuberculosis* was detected from the exudate of the left axillary lymph nodes but not from the sputum, and tuberculous lymphadenitis diagnosis was made. Thereafter, the patient was treated with isoniazid (300 mg), rifampicin (450 mg), pyrazinamide (1.5 g), and ethambutol (1 g) daily. On the 19th day of treatment, he complained of abdominal pain and diarrhea, and on day 20, palpable purpura appeared on both of his legs (Figure 1A). Laboratory findings were as follows: white blood cell count, 11.3 × 10^3^/μL; eosinophils, 0.2%; hemoglobin level, 13.2 g/dL; platelet count, 498 × 10^3^/μL; international normalized ratio, 1.05; D‐dimer, 61.9 μg/mL; percentage factor XIII activity, 77%; serum urea level, 20.9 mg/dL; creatinine level, 0.55 mg/dL; C‐reactive protein level, 12.5 mg/dL; serum, IgA 472 mg/dL; IgE, 122 IU/mL. Meanwhile, the results of serologic tests for antinuclear antibodies, rheumatoid factor, antineutrophil cytoplasmic antibodies, and hepatitis B and C viruses were all negative. Urinalysis showed a protein level of 50 mg/dL, and the patient tested negative for occult blood. Abdominal computed tomography showed wall thickening of the duodenum and small intestine, and mild to moderate ascites (Figure 2). An endoscopic examination demonstrated highly reddish erosions in the antrum of the stomach (Figure 3A) and circular ulcers in the descending part of the duodenum (Figure 3B). Biopsy of skin lesions showed leukocytoclastic vasculitis (Figure 1B). The deposition of IgA on the vascular wall was not proven, and we reached a diagnosis of IgAV in combination with palpable purpura and digestive symptoms.

**Figure 4:**
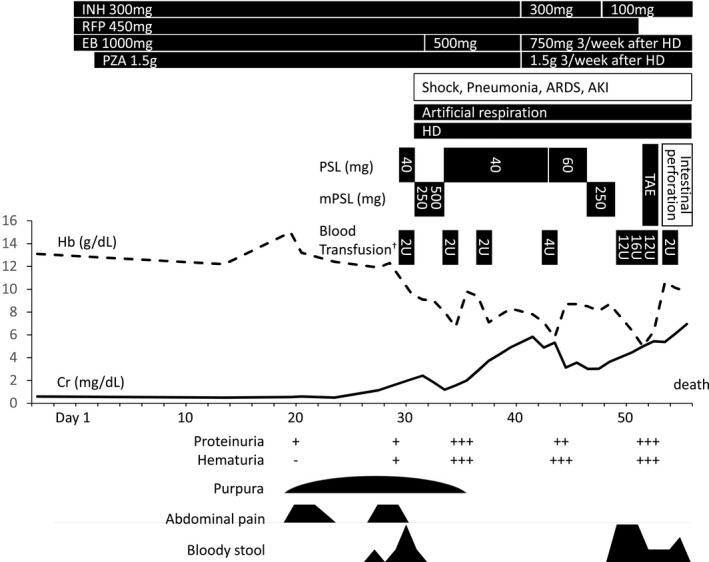
Clinical course after tuberculosis treatment was initiated. ^†^Units of red cell concentrate used for blood transfusion. AKI, acute kidney injury; ARDS, acute respiratory distress syndrome; Cr, creatinine; EB, ethambutol; Hb, hemoglobin; HD, hemodialysis; INH, isoniazid; mPSL, methylprednisolone; PSL, prednisolone; PZA, pyrazinamide; RFP, rifampicin; TAE, transcatheter arterial embolization.

The clinical course is shown in Figure 4. We considered corticosteroid administration to improve his abdominal symptoms, but we believed that this also carried a risk of worsening of tuberculosis and diabetes, so we opted to manage the patient with fasting and rest instead. A small amount of bloody stool was noted on day 27, but upper and lower gastrointestinal endoscopy did not reveal a definite source of bleeding. On day 30, the patient suffered hypovolemic shock as a result of massive blood loss, and we started systemic management, blood transfusion, and venous corticosteroid administration. Simultaneously, acute respiratory distress syndrome caused by aspiration pneumonia, and acute kidney injury occurred, which indicated that the patient required artificial respiration and hemodialysis. Hemorrhage was relieved temporarily, but a large amount of bloody stool reappeared on day 50, making it impossible to manage his condition by conservative treatment. Although we considered performing abdominal surgery, his general condition was deemed too severe for this approach. Therefore, abdominal angiography was performed with sufficient informed consent. Because the extravasation of contrast agent was observed in the jejunum, we performed transcatheter arterial embolization. Consequently, blood loss decreased, but gastrointestinal perforation merged. The patient died on day 56. His relatives did not accept the autopsy.

## 3 DISCUSSION

We report a patient who developed leukocytoclastic vasculitis during mycobacterial infection treatment. Leukocytoclastic vasculitis is a term for pathological finding, and several vasculitides show this finding. Among these vasculitides, IgAV was defined as vasculitis with IgA1‐dominant immune deposits that invades small blood vessels in the skin and gastrointestinal tract and often cause arthritis.^5^ However, IgA deposition cannot be proven in all cases, and some cases are difficult to diagnose.^2^ In the present case, there was no opportunity to prove IgA deposition. Biopsies from the gastric and duodenal mucosa did not contain appropriately sized blood vessels, and the involvement of vasculitis could not be pointed out. Therefore, we made the diagnosis of IgAV from clinical symptoms (ie, palpable purpura and abdominal pain) on the basis of the conventional criteria for Henoch‐Schönlein purpura proposed by the American College of Rheumatology (ACR) and the European League Against Rheumatism, Pediatric Rheumatology International Trials Organization, and Pediatric Rheumatology European Society (EULAR/PRINTO/PRES).^6^, ^7^, ^8^

The pathogenesis of IgAV is not yet clear, although several factors—such as infectious agents, dietary allergens, vaccines, and drugs—are presumed to be potential immunologic triggers. Tuberculosis has also been reported as an infectious agent associated with IgAV.^4^ The mechanism by which tuberculosis causes IgAV is unknown. Cutaneous vasculitis associated with tuberculosis is speculated to be caused by: (a) direct invasion of the vessel wall by tubercle bacilli; (b) immunological reaction involving the deposition of immune complexes; (c) intravascular release of mycobacteria followed by an Arthus reaction and delayed hypersensitivity response; or (d) rifampicin‐dependent antibody and immune complex formation.^9^

We searched for relevant English and Japanese articles in PubMed using the following keywords: IgA vasculitis, Henoch‐Schönlein purpura or leukocytoclastic vasculitis, and tuberculosis. Twenty‐six cases considered as IgAV met the classification criteria set by the ACR or the EULAR/PRINT/PRES^9^, ^10^, ^11^, ^12^, ^13^, ^14^, ^15^, ^16^, ^17^, ^18^, ^19^, ^20^, ^21^, ^22^, ^23^, ^24^, ^25^, ^26^, ^27^, ^28^, ^29^; these consisted of 14 cases before the treatment of tuberculosis (pretreatment group) and 12 cases after the treatment of tuberculosis (post‐treatment group) (Tables 1 and 2). The overall median age of patients was 37 years, and 19 (73.1%) of them were male. Gastrointestinal symptoms occurred in 5 (35.7%) of pretreatment patients and in 4 (33.3%) of post‐treatment patients. In the pretreatment group, tuberculosis treatment was performed in 10 cases (71.4%), and only 1 case was treated with steroids. In the post‐treatment group, antituberculous drugs were discontinued in 8 cases (66.7%), and steroids were administered in 7 cases (58.3%). In both groups, there was a single case of death, both of which were caused by exacerbation of the underlying disease, and the present case marks the first report of death caused by deterioration of digestive tract lesions. Because IgAV is thought to be caused by the immune response, the treatment of removing allergens is appropriate. Therefore, it is reasonable to start tuberculosis treatment for pretreatment patients and to cease treatment with antituberculosis drugs for post‐treatment patients. In the present case, cessation of treatment with antituberculosis drugs was considered, but because of concerns that this could lead to worsening of tuberculosis, we decided to continue with the treatment.

**Table 1:** Characteristics and outcomes in patients with IgA vasculitis complicated with tuberculosis (Onset before tuberculosis treatment)

Reference	Age (year)	Sex	Type of tuberculosis	Renal symptoms	Arthralgia/arthritis	Gastrointestinal symptoms	LCV	IgA deposit^a^	Treatment	Outcome
Dalgleish and Ansell	44	M	Pulmonary tuberculosis	+	−	+	NR	NR	Supportive therapy	Survive
	31	F	Pulmonary tuberculosis	+	−	+	NR	NR	Supportive therapy	Death
	47	M	Pulmonary tuberculosis	−	+	−	NR	NR	Supportive therapy	Survive
Washio et al	21	M	Tuberculous pleurisy	+	−	−	NR	+	Tuberculosis treatment	Survive
Sais et al	61	F	Tuberculous lymphadenitis	−	−	−	+	+	Tuberculosis treatment, Colchicine	Survive
Lee et al	15	F	Pulmonary tuberculosis	−	+	−	+	NR	Tuberculosis treatment	Survive
	13	F	Positive for blood mononuclear cell PCR	−	−	+	+	NR	Tuberculosis treatment	Survive
Minguez et al	36	M	Pulmonary tuberculosis, Tuberculous pleurisy	−	+	−	+	NR	Tuberculosis treatment, NSAIDs	Survive
Islek et al	8	F	Pulmonary tuberculosis	−	−	+	NR	NR	Tuberculosis treatment	Survive
Kim et al	49	M	Tuberculous lymphadenitis	−	+	−	+	NR	Tuberculosis treatment	Survive
Isobe et al	54	M	Pulmonary tuberculosis	+	−	−	+	+	Tuberculosis treatment, Systemic corticosteroid	Survive
Carvalho et al	50	M	Pulmonary tuberculosis	−	+	−	+	NR	NSAIDs, antihistamines	Survive
Bueno Filho et al	45	F	Tuberculous lymphadenitis	+	−	−	+	+	Tuberculosis treatment	Survive
Meziane et al	19	M	Pulmonary tuberculosis, Tuberculous pleurisy, Anal tuberculosis	−	+	+	+	−	Tuberculosis treatment	Survive

Abbreviations: F, female; LCV, leukocytoclastic vasculitis; M, male; NR, not reported.

^a^ IgA deposition in the vessels of skin or kidney.

**Table 2:** Characteristics and outcomes in patients with IgA vasculitis complicated with tuberculosis (Onset after tuberculosis treatment)

Reference	Age (year)	Sex	Type of tuberculosis	Antituberculosis drugs	Interval^a^(days)	Renal symptoms	Arthralgia/arthritis	Gastrointestinal symptoms	LCV	IgA deposit^b^	Treatment	Outcome
McLachlan	34	M	Tuberculous pericarditis, Tuberculous pleurisy, Pulmonary tuberculosis	SM, INH	20	+	+	+	NR	NR	Systemic corticosteroid, ACTH	Survive
	21	M	Pulmonary tuberculosis	SM, INH	>400	+	+	+	NR	NR	Withdrawal of antituberculosis drugs, Systemic corticosteroid	Survive
Chan et al	64	M	Pulmonary tuberculosis, Tuberculous pleurisy	INH, RFP, PZA, EB	150	+	−	−	+	+	Withdrawal of antituberculosis drugs	Survive
	41	M	Pulmonary tuberculosis	INH, RFP, PZA, EB	120	+	−	−	+	+	Withdrawal of antituberculosis drugs	Death
Mishima et al	34	M	Pulmonary tuberculosis, Tuberculous pleurisy	INH, RFP, SM	150	−	+	−	NR	NR	Systemic corticosteroid	Survive
Han et al	41	M	Disseminated tuberculosis	Unknown	15	+	−	−	+	+	Tuberculosis treatment	Survive
Kitamura et al	38	M	Pulmonary tuberculosis	INH, RFP, PZA, EB	90	+	+	+	+	+	Systemic corticosteroid	Survive
Chanprapaph et al	62	M	Pulmonary tuberculosis	INH, RFP, EB	14	−	−	−	+	+	Withdrawal of antituberculosis drugs, Topical corticosteroid, Antihistamines	Survive
Bhatia et al	14	M	Disseminated tuberculosis	INH, RFP, PZA, EB	42	−	−	−	+	−	Withdrawal of antituberculosis drugs, Systemic corticosteroid	Survive
AVCU	12	F	Pulmonary tuberculosis	INH, RFP, PZA, EB	29	−	−	−	+	NR	Withdrawal of antituberculosis drugs, Systemic corticosteroid, Antihistamines	Survive
Gargouri et al	29	M	Pulmonary tuberculosis	INH, RFP, PZA, EB	3	−	+	−	+	+	Withdrawal of antituberculosis drugs	Survive
Shim and Jung	72	M	Pulmonary tuberculosis	INH, RFP, EB	14	−	−	+	+	−	Withdrawal of antituberculosis drugs, Systemic corticosteroid	Survive
This case	71	M	Tuberculous lymphadenitis	INH, RFP, PZA, EB	19	+	−	+	+	NR	Systemic corticosteroid	Death

Abbreviations: EB, ethambutol; F, female; INH, isoniazid; LCV, leukocytoclastic vasculitis; M, male; NR, not reported; PZA, pyrazinamide; RFP, rifampicin; SM, streptomycin.

^a^ Interval between the start of tuberculosis treatment and onset of symptoms of IgA vasculitis.

^b^ IgA deposition in the vessels of skin or kidney.

Gastrointestinal symptoms of IgAV include abdominal pain, nausea, vomiting, and occult or overt intestinal bleeding.^1^, ^2^, ^3^ The small intestine is the most frequently involved site in the gastrointestinal tract. The descending part of the duodenum is characteristically involved more often than the bulb. Endoscopic findings exhibit various forms such as diffuse mucosal redness, petechiae, hemorrhagic erosions, and ulcers.^30^ Although symptomatic treatment alone often improves symptoms of IgAV, corticosteroids are useful in cases with severe abdominal symptoms. Corticosteroids should be considered early in cases with severe abdominal symptoms, because early corticosteroid therapy has been reported to reduce the need for abdominal surgery.^31^ We delayed the use of corticosteroids for fear of worsening the course of tuberculosis, but this was not necessary because corticosteroids have also been reported to improve the prognosis of tuberculosis.^32^ In addition, we could have used higher doses of corticosteroids because rifampicin has been reported to reduce the effectiveness of corticosteroids.^33^ On the other hand, deaths caused by infections associated with the use of glucocorticoids have been reported in older adults with IgAV, and it is thought that their use requires careful attention.^34^

In summary, there are only a few reports of IgAV complicated with tuberculosis, and the case we have presented marks the first fatal case attributed to gastrointestinal tract lesions. If IgAV caused by antituberculosis drugs is suspected, discontinuation of tuberculosis treatment is usually considered, but in cases marked by severe abdominal pain or gastrointestinal bleeding, use of corticosteroids should be considered. There is no need to withhold the use of corticosteroids because of the coexistence of tuberculosis. On the contrary, patients taking rifampicin may usually need more steroids. Careful attention should be paid when administering steroids to elderly patients with IgAV, as deaths from infections have been reported.

## CONFLICT OF INTEREST

The authors declare that there is no conflict of interest regarding the publication of this article.

## AUTHOR CONTRIBUTIONS

NY: wrote the manuscript. ST, SK, AO, MK, TA, TM, JY and AM: revised the manuscript. SF: supervised the final draft.
